# Tandem Repeats and G-Rich Sequences Are Enriched at Human CNV Breakpoints

**DOI:** 10.1371/journal.pone.0101607

**Published:** 2014-07-01

**Authors:** Promita Bose, Karen E. Hermetz, Karen N. Conneely, M. Katharine Rudd

**Affiliations:** 1 Department of Human Genetics, Emory University School of Medicine, Atlanta, Georgia, United States of America; 2 Department of Biostatistics and Bioinformatics, Emory University School of Public Health, Atlanta, Georgia, United States of America; Florida State University, United States of America

## Abstract

Chromosome breakage in germline and somatic genomes gives rise to copy number variation (CNV) responsible for genomic disorders and tumorigenesis. DNA sequence is known to play an important role in breakage at chromosome fragile sites; however, the sequences susceptible to double-strand breaks (DSBs) underlying CNV formation are largely unknown. Here we analyze 140 germline CNV breakpoints from 116 individuals to identify DNA sequences enriched at breakpoint loci compared to 2800 simulated control regions. We find that, overall, CNV breakpoints are enriched in tandem repeats and sequences predicted to form G-quadruplexes. G-rich repeats are overrepresented at terminal deletion breakpoints, which may be important for the addition of a new telomere. Interstitial deletions and duplication breakpoints are enriched in *Alu* repeats that in some cases mediate non-allelic homologous recombination (NAHR) between the two sides of the rearrangement. CNV breakpoints are enriched in certain classes of repeats that may play a role in DNA secondary structure, DSB susceptibility and/or DNA replication errors.

## Introduction

Genomic CNV is a major cause intellectual disability, autism spectrum disorders, epilepsy, and psychiatric disorders. Large, pathogenic CNVs are located throughout the human genome and include tens to hundreds of genes [1], [2]. Though most germline CNVs have different chromosome breakpoints [3]–[6], it is possible that breakpoint regions share common DNA features that make them susceptible to double-strand breaks (DSBs). This is true of chromosome rearrangements in leukemia that vary in location, but share repetitive DNA and/or DNAse hypersensitive sites at breakpoint cluster regions (BCRs) [7]–[9].

Classic studies of chromosomal fragile sites have revealed that DNA sequence and structure can influence chromosome breakage. Fragile sites were originally identified as breaks and gaps in metaphase chromosomes, induced under conditions of DNA replication stress [10], [11]. Mapping and sequence analyses of fragile sites have uncovered repetitive classes of DNA at many loci. Trinucleotide repeats and tandemly repeated minisatellites underlie many rare fragile sites [12], [13]. The FRA7E and FRA16D common fragile sites are made up of AT-rich repeats [14], and yeast studies have shown that the FRA16D AT-rich dinucleotide repeat intrastrand pairs to form a secondary structure that stalls DNA replication [15]. Though there is no single DNA sequence responsible for fragile sites in the human genome, in general fragile sites are made up of repetitive DNA that may form secondary structures.

Some studies of germline CNV breakpoints have attempted to identify common DNA sequences that, like fragile sites, contribute to chromosome breakage [16]–[21], but the search for breakage-prone DNA at CNV boundaries is challenging for a number of reasons. First, more than half of the human genome is made up of repetitive DNA, so finding a repeat at or near a CNV breakpoint may be a circumstantial finding unrelated to breakage. Second, DNA resection after chromosome breakage can lead to a CNV breakpoint that is kilobases (kb) away from the initial DSB [22], [23]. Thus, studies that only focus on the sequence directly adjacent to the post-repair junction will miss some DSB sites. Finally, since chromosome breaks are caused by heterogeneous factors, it is necessary to analyze breakpoints from a large cohort of annotated CNVs to find DNA motifs that are significantly enriched at breakpoints.

Using our large dataset of fine-mapped and sequenced CNV breakpoints from patients with neurodevelopmental disorders, we applied several motif and repeat discovery tools to search for DNA sequences enriched at CNV breakpoints compared to control regions of the genome. We broadened the breakpoint regions to include flanking sequence and account for DNA resection. To search for a common breakage-associated motif, we analyzed patient breakpoint regions and control sequences using Multiple EM for Motif Elicitation (MEME) and nested motif independent component analysis (NestedMICA). We also searched for repetitive DNA with Tandem Repeats Finder, QuadParser, and RepeatMasker. This large-scale analysis revealed an enrichment of tandem repeats and potential G-quadruplex sequences at human CNV breakpoints, providing insight into DNA sequences susceptible to DSBs.

## Materials and Methods

### CNV breakpoint and control sequences

We previously fine-mapped and/or sequenced CNV breakpoints from 116 individuals with abnormal clinical cytogenetic testing results [5], [6]. Patients have unique deletions and duplications that alter the copy number of different genes, so they do not share a common phenotype. In general, individuals with large pathogenic CNVs exhibit developmental delay, intellectual disability, autism spectrum disorders, and/or congenital anomalies. We analyzed 48 terminal deletions, 41 inverted duplications adjacent to terminal deletions, 11 translocations, 10 interstitial deletions, four interstitial duplications and two terminal duplications. Terminal deletions and terminal duplications have one breakpoint per rearrangement. Translocations, interstitial deletions and interstitial duplications have two breakpoints per rearrangement. For 18q-71c's translocation, we only identified the chromosome 18 breakpoint; the other breakpoint on chromosome 4 is cryptic [5]. Thus, there are a total of 140 breakpoints in 116 individuals.

We calculated 4-kb windows surrounding 140 CNV breakpoints and downloaded the corresponding DNA sequence from the NCBI 36.1/hg18 build of the human genome assembly using the Table Browser from the UCSC Genome Browser (http://genome.ucsc.edu/). Four-kb CNV breakpoint regions were unique and did not overlap with one another. We also randomly selected 4-kb control sequences from the same genome build. We concatenated the coordinates of chromosomes 1–22, X and Y that make up the 3,080,419,480 bp in the haploid human genome. Next we used a random number generator to select 10,000 numbers between one and 3,080,419,480. We added four kb to each number to produce start and stop coordinates for 10,000 regions and downloaded the associated DNA sequences from the UCSC Genome Browser. We excluded control regions with “N” bases that correspond to sequencing gaps, resulting in 9243 ungapped 4-kb sequences. From these, we randomly selected 140 sequences 20 times to make up 20 datasets of 140 control sequences. We saved 140 CNV breakpoint sequences and 140 control sequences per dataset in FASTA format for analysis.

### MEME and NestedMICA

We searched for common motifs in the CNV breakpoint and 20 control datasets using MEME [24] and NestedMICA [25] with default parameters to find a single ungapped 50-basepair (bp) motif. We ran NestedMICA motif inference tool (NMinfer) and NestedMICA motif scanner module (NMscan) with a cutoff of −15 to determine the number of motifs per sequence. MEME and NestedMICA programs were executed on the Emory Human Genetics Computing Cluster (HGCC). We used NMinfer to identify the 50-bp NestedMICA motif in all 21 datasets and MochiView [26] to align the motifs to the sequences in the CNV breakpoint and control datasets.

### Repeat searches

To identify *Alu* repeats in the CNV breakpoint and control datasets, we ran RepeatMasker using default settings [27]. We identified tandem repeats and G-quadruplex sequences using Tandem Repeats Finder (TRF) [28] and QuadParser [29], respectively. All three programs were executed with default parameters. We used custom scripts to calculate the lengths of non-overlapping tandem repeats. We calculated the GC content of sequences using the geecee program (http://mobyle.pasteur.fr/cgi-bin/portal.py#forms::geecee) within the European Molecular Biology Open Software Suite (EMBOSS) [30].

We used chi-squared goodness-of-fit tests to test whether each type of repeat was proportionally distributed across CNV types (i.e., independent of CNV type). We used two-sided binomial tests to test each type of CNV breakpoint for enrichment with each type of repeat. Since we performed 18 of these enrichment tests (testing six CNV types for enrichment for three repeat types), we performed Bonferroni adjustment for the 18 tests and used a p-value cutoff of .00278 = .05/18 to assess significance.

## Results

### Human CNV breakpoints

Our goal was to identify DNA sequence motifs that are overrepresented in human CNV breakpoint regions compared to control regions of the genome. We analyzed the breakpoint sequences of pathogenic CNVs ascertained from 116 children with phenotypes including intellectual disability, developmental delay, congenital abnormalities, and autism spectrum disorders. We excluded recurrent CNVs mediated by NAHR between segmental duplications. The 140 breakpoints from 116 CNVs have been fine-mapped by high-resolution array comparative genome hybridization (CGH) [5], [6]. Thirty-two out of 116 CNV junctions have been sequenced, resolving the breakpoints to the bp. Most of the sequenced junctions were simple with little or no microhomology at the breakpoint junctions; three had more complex junctions with short insertions 10–16 bp long [5], [6] (Table S1). The other 84 CNV junctions were fine-mapped with custom microarrays that had, on average, one oligonucleotide probe per 200 bp. Oligonucleotide spacing is not uniform throughout the genome due to repetitive sequences that confound unique probe design. Thus, the mean and median resolutions of breakpoints are 468 bp and 101 bp, respectively (Table S1).

To characterize a diverse collection of CNV breakpoints, we included breakpoints from 48 terminal deletions, 41 inverted duplications adjacent to terminal deletions, 11 translocations, 10 interstitial deletions, four interstitial duplications and two terminal duplications. Interstitial deletions and duplications have two breakpoints in the same chromosome arm, and translocations have two breakpoints in different chromosomes. Terminal deletions and duplications have a single breakpoint. Inverted duplications adjacent to terminal deletions are a specific type of CNV where the deletion and duplication form as part of one chromosome rearrangement. In this case, the terminal deletion is the site of the initial DSB [5], [6], so we only included that breakpoint in the analysis. Since our CNV dataset is enriched in terminal deletions and duplications, breakpoints are overrepresented towards chromosome ends. The mean and median distances from the CNV breakpoint to the end of the chromosome are 4.8 Mb and 2.3 Mb, respectively (Table S2). It is important to recognize that all CNVs in this study extend beyond the terminal segmental duplications that make up human subtelomeres. Thus, none of the CNV breakpoints lie in subtelomeric segmental duplications.

Array CGH and junction sequencing can resolve chromosome breakpoints to a relatively small region; however, that may not correspond to the exact DSB site. After the initial DSB, 5′ to 3′ DNA resection can lead to a CNV breakpoint that is up to 1.3 kb away [22], [23]. We included additional sequence around each of the 140 breakpoints to account for DNA resection and array resolution. Breakpoint regions are based on the normal locus in the reference genome (before breakage), not the patient's CNV (after breakage). In the case of sequenced breakpoint junctions, we added sequence two kb proximal and two kb distal of the breakpoint. For breakpoints that were resolved by array but not sequencing, we added two kb proximal and distal to the midpoint between the abnormal and normal probes that defined the CNV breakpoint. Thus, each breakpoint region is four kb and centered around the post-repair chromosome breakpoint. We downloaded the 140 4-kb breakpoint regions from the reference genome assembly (NCBI Build 36.1/hg18).

As a comparison, we analyzed 4-kb control regions from the human genome. We compiled 140 control sequences 20 times to make up 20 control datasets (see Methods). In the following experiments, we compared the motifs in the CNV breakpoint dataset to those in the 20 control datasets. We applied motif-finding tools to search for DNA motifs with the potential to form secondary structures susceptible to DSBs. Since most of our breakpoints are fine-mapped, but not sequenced, we focused on long repeats that span much of the breakpoint region. We did not analyze short motifs reported at other chromosome breakpoints (e.g., 6-8-bp translin target sites) due to the imprecision of most CNV junctions in our study.

### Common motif search

**Figure 1:**
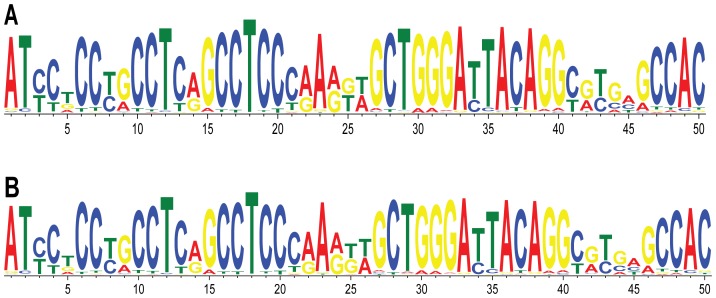
Common 50-bp motif in CNV breakpoint dataset. Logo plots for (**A**) MEME and (**B**) NestedMICA motifs show nearly identical consensus sequences.

**Figure 2:**
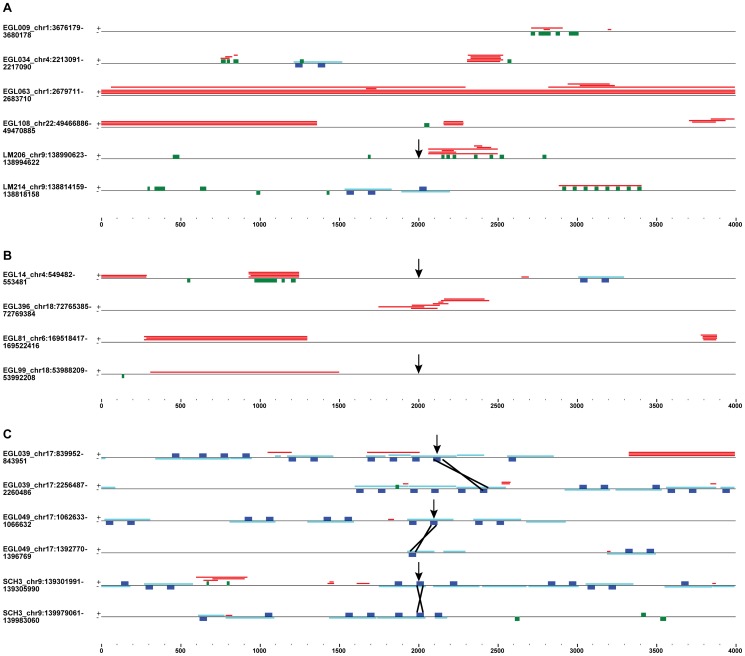
Repeats in 4-kb CNV breakpoint regions. The location of tandem repeats (red), G-quadruplexes (green), *Alu*s (light blue) and 50-bp motif (dark blue) sequences are shown for a subset of terminal deletion (**A**), inverted duplication adjacent to terminal deletion (**B**) and interstitial deletion (**C**) breakpoint regions. Repeats on the positive (+) and negative (−) strands are shown on the top and bottom of the black line, respectively. Arrows point to sequenced breakpoint junctions. The breakpoint region from EGL108 underlies terminal deletions and one interstitial duplication. Black Xs show *Alu*-*Alu* recombination sites for sequenced junctions (**C**).

It is possible that CNVs are caused by breakage in a common DNA sequence motif present in many or all CNV breakpoints. To look for common motifs among the 140 CNV breakpoints, we performed MEME [24] and NestedMICA [25] searches. We queried the top 50-bp motif in the CNV breakpoint dataset using default parameters for both programs. MEME and NestedMICA output almost identical motifs; only one bp is different between the two consensus sequences, at position 25 (Figure 1). We performed NestedMICA searches with the same parameters in the 20 control datasets and found motifs that were very similar to each other, and very similar to the motif present in the CNV breakpoint dataset (Figure S1). Further review of this common motif revealed that it is part of the *Alu* repeat sequence. This 50-bp motif is present twice in the ∼300-bp *Alu* consensus (Figure 2) and includes a 26-bp core sequence that is a hotspot within *Alu*s involved in gene rearrangements [31].

It is possible that although the 50-bp motif is present in both breakpoint and control datasets, it is enriched in CNV breakpoints. To explore this, we examined the number of motifs in CNV breakpoints and control regions. Using the 50-bp NestedMICA motif detected in the CNV breakpoint dataset, we visualized the motif alignments with MochiView [26] and counted the number of motifs in all 21 datasets. The number of 4-kb sequences with at least one motif is not enriched in the CNV breakpoint dataset (68) versus controls (56–85), and the total number of motifs in the CNV breakpoint dataset (161) is not greater than the number of motifs in the control datasets (137–209) (Figures S2 and S3, Table S3). Since *Alu*s are the most abundant mobile element in the human genome [32], it is not surprising that we find a substring of the *Alu* sequence as the most common 50-bp sequence in the CNV breakpoint dataset and the 20 control datasets; however, this motif is not overrepresented in breakpoint regions compared to control regions of the genome.

### Repeats enriched at CNV breakpoints

Diverse types of repetitive sequences may be involved in DSBs that give rise to CNVs. This is the case for fragile sites, which are made up of various classes of satellite DNA, dinucleotide and trinucleotide repeats. In addition, different types of repetitive DNA predicted to form secondary structures underlie many BCRs in tumor genomes [7]–[9], [16], [33], [34]. To investigate repetitive DNA sequences involved in germline CNVs, we searched for tandem repeats and predicted G-quadruplex DNA in the CNV breakpoint and control datasets.

**Figure 3:**
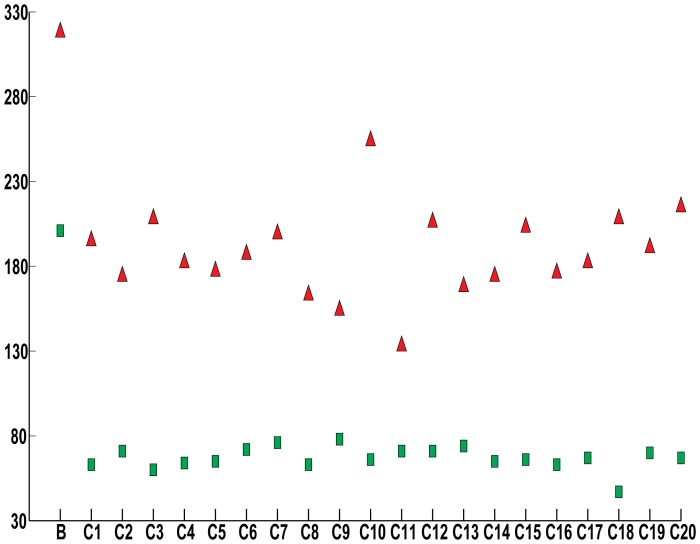
Number of repeats per dataset. The total number of tandem repeats (red triangles) and G-quadruplex (green rectangles), sequences per CNV breakpoint (B) and control (C1-C20) datasets are plotted.

We used Tandem Repeats Finder with default settings to identify tandem repeats in the 21 datasets. Tandem repeats are not based on a consensus sequence, rather they are defined by two or more duplicated sequences arrayed head-to-tail. Since tandem repeats may overlap one another (Figure 2), we counted the total number of tandem repeats as well as the total non-overlapping bp occupied by at least one tandem repeat in each 4-kb sequence. In the CNV breakpoint dataset, 104 out of 140 breakpoint sequences had at least one tandem repeat (Table S4). For the 104 sequences with tandem repeats, 25–4000 bp were occupied by tandem repeats, and the mean and median amounts of sequence including at least one tandem repeat were 330 bp and 133 bp, respectively. The 20 control datasets had 71–95 out of 140 sequences with at least one tandem repeat (mean  = 82; median  = 83). For those control sequences with at least one tandem repeat, the mean and median numbers of bp occupied by a tandem repeat per 4-kb sequence were 156 bp and 63 bp, respectively. Since all 21 datasets had the same number of bp analyzed (4 kb * 140 sequences  = 560 kb), we can compare the number of tandem repeats without adjusting for the size of the dataset. The CNV breakpoint dataset had 318 tandem repeats, whereas the control datasets had 133–254 tandem repeats (Figure 3). Thus, the CNV breakpoint regions are enriched in the number and density of tandem repeats compared to the control regions.

Tandem repeats include other classes of repetitive sequences, including triplet repeats and satellite DNA. It is possible that the enrichment in tandem repeats in the CNV breakpoint dataset is due largely to one particular type of duplicated sequence. Instead, we found that the tandem repeats in the breakpoint regions vary in repeat unit size, repeat array size, AT- and GC-content. We concatenated the tandem repeats in 104 CNV breakpoints to assemble the non-overlapping tandem repeat sequences and avoid counting segments of breakpoint regions more than once. Non-overlapping tandem repeat regions have mean and median GC percentages of 44% and 49%, respectively. Thus, both AT-rich and GC-rich tandem repeats are present at CNV breakpoints.

We also investigated sequences predicted to form G-quadruplexes in CNV breakpoint and control datasets. Sequences that contain four tracts of at least three guanines separated by other bases can form G-quadruplexes by intrastrand pairing between the four G-rich tracts. Such G-rich sequences can assemble G-quadruplex structures *in vitro* [35] and cause chromosome breakage and genomic instability *in vivo* [36], [37]. We searched for the G-quadruplex consensus sequence, G_3+_N_1–7_G_3+_N_1–7_G_3+_N_1–7_G_3+_, using the QuadParser program [29]. Sixty-eight out of 140 CNV breakpoint regions have at least one G-quadruplex, whereas 38 to 52 control regions in the 20 control datasets have at least one G-quadruplex (mean and median  = 42). There are 201 G-quadruplexes in the CNV breakpoint dataset and 47–78 G-quadruplexes in the control datasets (Figure 3; Table S5). Thus, CNV breakpoints are enriched in the G-quadruplex consensus sequence.

It is possible that the enrichment in G-quadruplex sequences in the CNV breakpoint datasets stems from an increase in overall GC-richness in CNV breakpoints. The human genome is organized into GC-rich and AT-rich isochores [38], and the genome-wide average of GC content is 41% [39]. The mean GC content of the 20 control datasets ranged from 39.8–41.6%, whereas the mean GC content of the CNV breakpoint regions was 47.2% (Table S6). CNV breakpoints are 133 kb to 75 Mb from the nearest telomere; the mean and median distances are 4.8 Mb and 2.3 Mb, respectively (Table S2). Thus, many breakpoints lie within the terminal chromosome band that is known to be elevated in GC content [40], [41]. Since terminal deletion and duplication CNV breakpoints lie closer to chromosome ends than other CNV breakpoints, we would expect them to be GC-rich. However, breakpoint regions from all six types of CNV had a higher GC percentage than the genome average (Table 1). Therefore, the GC enrichment in CNV breakpoints is not due to only a subset of terminal chromosome rearrangements.

**Table 1:** Repeats enriched and depleted in CNV breakpoints. The GC content and number of breakpoints are listed for the six CNV types.

CNV type	%GC	Breakpoints	Tandem repeats	G-quads	*Alu*s
**Terminal deletion**	50.1	48	122	99**	62
**Inverted duplication with terminal deletion**	43.6	41	70	39*	36*
**Translocation**	46.1	21	40	24	42
**Interstitial deletion**	50.0	20	60	30	72**
**Interstitial duplication**	45.4	8	25	9	28**
**Terminal duplication**	41.0	2	1	0	2
Total		140	318	201	242

The number of tandem repeats, G-quadruplexes, and *Alu*s per type of CNV are shown. Significant depletion (*) and enrichment (**) for repeats were determined by binomial test p-values <.00278 (.05/18, based on Bonferroni adjustment for 18 tests).

### Repeats enriched in different types of CNV

We analyzed the enrichment of tandem repeats, G-quadruplexes and *Alu*s in the breakpoints from the six types of CNVs (Table 1). Interstitial and terminal duplications are underrepresented in the 140 breakpoints (n = 10), whereas terminal deletions and inverted duplications adjacent to terminal deletions make up more than half of the 140 breakpoints. Tandem repeats, G-quadruplexes and *Alu*s were not distributed proportionally across CNV types according to chi-square goodness-of-fit tests (1e-15<p<.0024). Terminal deletion breakpoint regions have an average GC content of 50.1% and are enriched in G-quadruplexes (p = 9.2e-6). Inverted duplication terminal deletion breakpoints were slightly depleted for G-quadruplexes (p = .0019) and *Alu*s (p = 2.4e-7) (Table 1). Breakpoints from interstitial duplications and deletions were enriched in *Alu*s (p = 3.9e-10 and 3.0e-4, respectively). The enrichment of motifs at certain types of CNV breakpoints was striking and points to specific repetitive DNA being involved in various types of chromosome rearrangements.

## Discussion

Our analysis of 140 CNV breakpoints revealed an enrichment in tandem repeats and G-quadruplexes. It is possible that some of these sequences assemble secondary structures that are susceptible to DNA replication errors or DSBs. Tandem repeats have been described at other CNV breakpoints and are predicted to form a range of secondary structures [17]–[19]. In our CNV breakpoints, we find both AT-rich and GC-rich tandem repeats. Additional studies of DNA secondary structure and chromosome fragility are necessary to pinpoint the factors required for DSBs in particular classes of tandem repeats.

Breaks in repetitive sequences may facilitate particular types of chromosome rearrangements. DSBs that give rise to terminal deletions may be repaired by synthesis of a new telomere at the deletion breakpoint [5], [42]–[44]. Breaks that occur in or resect to G-rich DNA are ideal substrates for telomerase to prime a new telomere sequence, (5′-TTAGGG-3′)n. In addition, G-rich sequences with the ability to form secondary structures are susceptible to DSBs. Thus, sequences that underlie terminal deletion breakpoints may be G-rich due to the propensity for DSBs plus the likelihood of recovering a chromosome break repaired by a new telomere. We sequenced 13 of 48 terminal deletion junctions to pinpoint the post-repair CNV junction. None of the 13 terminal deletion junctions lies in a G-quadruplex or tandem repeat; however, LM206's chromosome 9q terminal deletion junction is 65 bp proximal of a cluster of G-rich tandem repeats and G-quadruplexes (Figure 2). It is tempting to speculate that a DSB in the G-rich repeat region resected 65 bp and was the site of telomere addition. In this case, the new telomere lies directly adjacent to a G-rich sequence at the breakpoint, 5′-GGGGCGGAGGGGCCGAAGCTGGCTGGTGG-3′ [5].

Though *Alu* repeats were not enriched in the entire CNV breakpoint dataset compared to control datasets, they were enriched in interstitial deletion and duplication breakpoint regions (Table 1). *Alu*s that recombine to form interstitial deletions and duplications are oriented in the same direction, share high sequence homology (typically >85% identical), and crossover at a homologous site within the *Alu*s [5], [45]–[48]. NAHR generates a hybrid *Alu* at the breakpoint that merges the two sides of the CNV, which is detectable by breakpoint sequencing. We sequenced three of the ten interstitial deletion junctions and none of the four interstitial duplication junctions. Sequence analysis revealed that EGL039, EGL049 and SCH3 breakpoints are the product of recombination between two highly identical *Alu* repeats [5] (Figure 2). In all three cases, the sequenced *Alu*-*Alu* breakpoints lie within a 50-bp motif. Other interstitial deletion breakpoints may have *Alu*s nearby, but are not the product of *Alu*-*Alu* NAHR. EGL094's sequenced interstitial deletion junction is the product of non-homologous end-joining (NHEJ) between two breakpoints that are not in *Alu*s [5]. However, EGL094's proximal breakpoint region has a cluster of five *Alu*s in four kb (Figure S2). Other studies of chromosome breakpoints have also found an enrichment of *Alu*s at interstitial deletion junctions [16], [20].

CNV breakpoint regions in our study were significantly more GC-rich than the genome average. A previous study of germline and somatic breakpoints suggested that deletion breakpoints were AT-rich, whereas translocation breakpoints were GC-rich [16]. The deletion and translocation breakpoints in our study are both GC-rich, with GC contents of 50% and 46%, respectively (Table 1). This difference is likely due to the chromosome rearrangements selected for the two studies: Abeysinghe *et al*. examined a large cohort of mostly somatic chromosome rearrangements, whereas our study included only germline chromosome rearrangements. In addition, 87/140 (87%) of the CNV breakpoints in our study occur in the last 10 Mb of chromosomes, which are more GC-rich than the genome average. There are likely different biases in GC content for deletions, depending on the origin of the deletion (germline vs. somatic) and the location of the deletion breakpoints. In their large-scale analysis of 663,446 breakpoints from diverse cancer genomes, De and Michor found an enrichment of tandem repeats and *Alu*s [34]. Thus, some classes of DNA repeats are shared between germline and somatic breakpoints.

Repeat density may also play a role in chromosome breakage. For example, both of EGL039's breakpoints are made up of several *Alu*s and tandem repeats. EGL063's terminal deletion breakpoint is entirely covered by tandem repeats across the 4-kb region, and LM206's terminal deletion breakpoint has overlapping tandem repeats and G-quadruplexes (Figure 2). In some cases, G-quadruplexes are part of the tandem repeat structure, rather than separate sequences (see EGL99 and LM214 breakpoints). In other breakpoints, different types of repeats are dispersed across the 4-kb region (see EGL034, EGL108 and EGL81 breakpoints). Repeats at CNV breakpoints could have an additive effect, whereby more repeats lead to a greater propensity for chromosome breakage and/or recombination. On the other hand, there may be only one repeat per locus that is responsible for chromosome rearrangement.

Our analysis of CNV breakpoint regions revealed an enrichment in tandem repeats and sequences predicted to form G-quadruplexes. Furthermore, particular classes of repeats are overrepresented at breakpoints of different types of CNV. Thus, when interpreting mechanisms of CNV formation, it is important to consider the DNA at breakpoints as well as the resulting chromosome rearrangement. Functional analysis of individual DNA motifs will delineate the sequences responsible for gross chromosomal rearrangement [49]–[51]. In addition, motif mining of even larger CNV breakpoint datasets from diverse CNV classes will tell us more about the factors required for CNV formation.

## Supporting Information

Figure S1 **Logo plots of top 50-bp motif detected in each control dataset (C1-C20) by NestedMica.**
(TIF)

Figure S2 **Repeats in 4-kb CNV breakpoint regions (html).** The location of tandem repeats (red), G-quadruplexes (green), *Alu*s (light blue) and 50-bp motif (dark blue) sequences are shown for each of the 140 CNV breakpoint regions.
(EPS)

Figure S3 **Repeats in 4-kb control regions (html).** 140 control regions from control dataset C3 are shown as an example of repeat content in control sequences.
(EPS)

Table S1 **CNV breakpoint resolution.** For 140 breakpoint regions, minimum, maximum, mean, and median breakpoint resolution is 0 bp, 7,542 bp, 468 bp, and 101 bp, respectively. The method of breakpoint mapping and the type of rearrangement is described. Insertion length is listed for three sequenced junctions with insertions.
(XLSX)

Table S2 **Distance from breakpoint region to the nearest chromosome end.**
(XLSX)

Table S3 **50-bp NestedMica (NM) motifs in the CNV breakpoint region dataset (B) and control datasets (C1-C20).** The number of sequences with at least one NM motif and the number of NM motifs per dataset are listed.
(XLSX)

Table S4 **Tandem repeats (TR) in the CNV breakpoint region dataset (B) and control datasets (C1-C20).** The number of sequences with at least one TR and the number of TRs per dataset are listed.
(XLSX)

Table S5 **G-quadruplex consensus sequences (G_3+_N_1–7_G_3+_N_1–7_G_3+_N_1–7_G_3+_) in the CNV breakpoint region dataset (B) and control datasets (C1-C20).** The number of sequences with at least one G-quadruplex and the number of G-quadruplexes per dataset are listed.
(XLSX)

Table S6 **GC content of CNV breakpoint region dataset (B) and control datasets (C1-C20).** For each dataset, minimum, maximum, mean, and median percent GC were calculated.
(XLSX)
