# Supplementary figures and images for: Tandem Repeats and G-Rich Sequences Are Enriched at Human CNV Breakpoints
Source: PLoS One. 2014 Jul 1;9(7):e101607. doi: 10.1371/journal.pone.0101607 (PMC4090240; doi:10.1371/journal.pone.0101607)

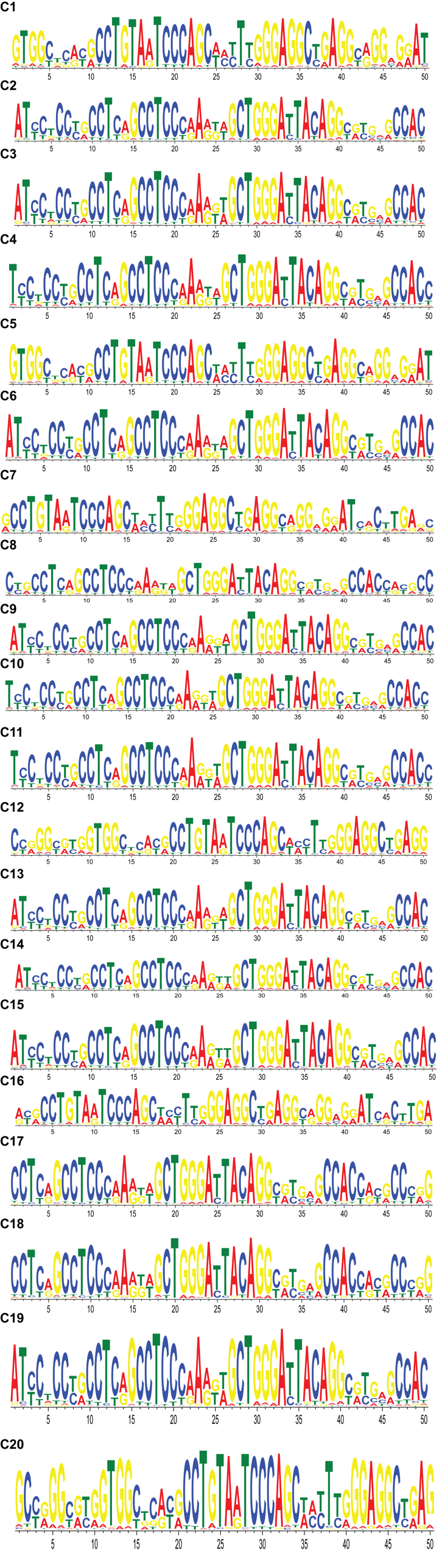

Supplement: Figure S1 — Logo plots of top 50-bp motif detected in each control dataset (C1-C20) by NestedMica. (TIF) [file pone.0101607.s001.tif]
